# Densification Behavior and Microstructure of Nickel Aluminum Bronze Alloy Fabricated by Laser Powder Bed Fusion

**DOI:** 10.3390/ma19010208

**Published:** 2026-01-05

**Authors:** Yizhe Huang, Guanjun Fu, An Wang, Zhongxu Xiao, Jinfeng Sun, Jun Wang, Xiaojia Nie

**Affiliations:** 1 Hubei Key Laboratory of Modern Manufacturing Quality Engineering, School of Mechanical Engineering, Hubei University of Technology, Wuhan 430068, China; yizhehuang@hbut.edu.cn (Y.H.); fuguanjun@hbut.edu.cn (G.F.); 102500010@hbut.edu.cn (A.W.); xiaozhongxu@hbut.edu.cn (Z.X.); 20051043@hbut.edu.cn (J.S.); junwang@hbut.edu.cn (J.W.); 2 Naval University of Engineering, Wuhan 430033, China

**Keywords:** laser powder bed fusion, nickel–aluminum–bronze alloy, densification behavior, microstructure, mechanical property

## Abstract

Nickel–Aluminum–Bronze (NAB) has gained significant attention in marine applications due to its excellent corrosion resistance and has shown growing potential for laser powder bed fusion (L-PBF) additive manufacturing. However, research on the fabrication of NAB alloys using L-PBF remains relatively limited. In this study, fully dense NAB samples were successfully fabricated through L-PBF process parameter optimization. The microstructural evolution and mechanical properties of both as-built and annealed L-PBF samples were systematically investigated and compared with those of traditionally cast NAB. The results reveal that the as-built L-PBF specimens primarily consist of columnar β′ grains, with the α phase distributed along the grain boundaries and a small amount of κ phase precipitated within the β′ matrix, distinctly different from the cast microstructure characterized by a columnar α-phase matrix with precipitated β′ and κ phases. After annealing at 675 °C for 6 h, the β′ phase in both methods decomposed into α + κ phases, and the original columnar structure in the L-PBF specimens transformed into a dendritic morphology. Compared to the cast samples, the L-PBF-produced NAB alloy exhibited significantly enhanced yield strength, tensile strength, and microhardness, attributable to rapid solidification during the L-PBF process. Following annealing, the yield strength and elongation increased by 12.8% and 184.4%, respectively, compared to the as-built condition, resulting from the decomposition of the martensitic phase into α + κ phases and further grain refinement.

## 1. Introduction

NAB alloys are the material of choice for critical marine infrastructure, such as pipeline valves and propulsion systems, due to their exceptional resistance to seawater corrosion and high mechanical robustness [1,2]. However, conventional manufacturing techniques, such as casting and forging, often struggle to produce components with complex geometries [3,4]. Furthermore, these traditional methods are susceptible to metallurgical defects, including coarse elemental segregation and porosity, which inevitably degrade the durability and performance of the alloy in harsh marine environments [5].

Additive manufacturing (AM) has emerged as a transformative solution for fabricating intricate structures that are difficult to achieve via subtractive methods [6]. Currently, Wire Arc Additive Manufacturing (WAAM) and Laser Powder Bed Fusion (L-PBF) are the predominant AM technologies employed for NAB alloys [7,8]. WAAM is characterized by high deposition rates and steep thermal gradients, which facilitate rapid solidification and result in a finer dendritic microstructure compared to casting, thereby enhancing mechanical strength [9,10,11]. Research by Cai et al. [12] indicated that the cyclic thermal history during the layer-by-layer WAAM deposition helps eliminate residual stresses and promotes grain growth, leading to substantial improvements in both yield strength and elongation compared to as-cast conditions. Nevertheless, precise control of process parameters is critical; excessive heat input can diminish the uniformity of the κ phase distribution, resulting in reduced hardness and geometric shrinkage [13]. To address these issues, Aliyu et al. [14] demonstrated that post-process aging at 675 °C could optimize mechanical properties. However, despite the economic benefits of high wire utilization and deposition efficiency (>1 kg/h), WAAM remains limited by inadequate dimensional accuracy and significant microstructural heterogeneity, which restricts its applicability for manufacturing high-precision components [15].

Compared to WAAM, L-PBF demonstrates compelling advantages for the precision manufacturing of NAB alloys [16,17]. Its competitiveness arises from exceptional adaptability to intricate geometries, high-fidelity replication of fine structural details, and superior dimensional accuracy. These attributes collectively enable the fabrication of complex seawater pump/valve components with stringent performance requirements [18,19,20]. Han et al. [21] maintained a volumetric energy density (VED) at 200–250 J/mm^3^ during L-PBF processing, fabricating NAB specimens exceeding 98.5% relative density. Microstructural analysis revealed β1′ martensite containing nano-twins and nanoscale κ-phase precipitates, contributing to enhanced mechanical properties: a microhardness of 386 HV and an ultimate tensile strength of 671 MPa. Hyatt et al. [22] further demonstrated the superior performance in L-PBFed NAB, particularly for low-energy-input specimens exhibiting martensitic structures. These microstructures significantly improved hardness and corrosion resistance. However, the rapid solidification characteristics intrinsic to L-PBF also generated substantial residual stresses, which adversely affected ductility.

Further research indicates that the mechanical properties of NAB alloys fabricated by L-PBF can be optimized through subsequent heat treatment. Murray et al. [23] fabricated high-density NAB specimen and annealing it at 700 °C for 1 h resulted in excellent corrosion resistance but severely limited plasticity, with elongation merely reaching 0.4%. Research by Dharmend et al. indicates that the ductility of the additively manufactured NAB alloy is significantly enhanced following a 6 h heat treatment at 675 °C [24]. Similarly, Shen et al. [25] demonstrated that appropriate quenching and tempering heat treatment can effectively reduce the anisotropy of NAB alloys following additive manufacturing while enhancing their mechanical properties. Xu et al. [26] further observed that quenching and aging treatment can mitigate the anisotropy of NAB alloys by promoting the coarsening of κ-phase structures, thereby enhancing the comprehensive mechanical properties of these alloys.

Although previous studies have reported the preparation of nickel–aluminum–bronze (NAB) alloys by L-PBF technology, the investigation of the microstructure evolution of L-PBF-fabricated NAB alloys remains limited. Particularly, the formation and stability of the microstructure dominated by β′ martensite under rapid solidification conditions, as well as its phase transformation behavior during post-processing heat treatment, have not been systematically clarified. Moreover, the relationship between densification behavior, phase composition (β′, α, and κ phases), and mechanical properties in L-PBF fabricated NAB alloys has not been fully elucidated.

Therefore, this study aims to systematically investigate the densification behavior, microstructure evolution, and mechanical properties of near-NAB alloys fabricated by L-PBF technology. A processing window close to full density was established through parameter optimization, and the microstructure and phase evolution of L-PBF fabricated NAB alloys were studied and compared with those of cast alloys. By correlating phase transformation and microstructure characteristics with mechanical properties, a deeper understanding of L-PBF-fabricated NAB alloys was provided.

## 2. Experimental and Methods

### 2.1. Materials

**Figure 1:**
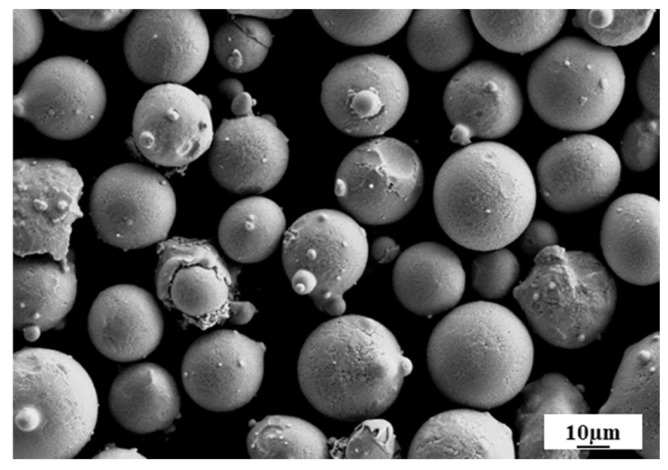
SEM image of NAB powder.

Gas-atomized NAB powder, supplied by Hunan Hengji Powder Technology Co., Ltd. (Yueyang, China), was produced from Cu-9Al-4Ni-4Fe-1Mn (C95800) die-casting rods. The morphology of NAB alloy powder is shown in Figure 1. The chemical composition of the received powder, as determined by Inductively Coupled Plasma Optical Emission Spectroscopy (ICP-OES), is presented in Table 1 alongside the standard composition of C95800. It is noted that while trace impurities (such as Si, Zn, and Pb) may exist, their cumulative content is negligible (<0.05 wt%) and the powder strictly adheres to the purity requirements of the standard. The particle size distribution of the powder is characterized by the cumulative percentiles of D_10_ = 25.89 μm, D_50_ = 38.9 μm, and D_90_ = 57.98 μm.

### 2.2. Specimen Preparations

The as-built specimens were prepared using a LiM-X260A L-PBF system (Tianjin L-PBF Laser Co., Ltd., Tianjin, China). The experimental procedures were divided into two phases: process optimization and performance characterization. The detailed experimental design matrix is presented in Table 2. In the first phase, to establish the processing window, the laser power (*P*) was varied from 300 W to 400 W, and the scanning speed (*V*) ranged from 500 mm/s to 1600 mm/s, resulting in 21 distinct parameter combinations. Based on the densification results, the optimal parameters (*P* = 300 W, *V* = 1000 mm/s) were selected for fabricating the specimens used in subsequent microstructural and mechanical characterizations. Fixed processing parameters included a hatch spacing of 0.11 mm and a layer thickness of 30 µm, which were determined based on the equipment manufacturer’s recommendations for copper alloys and preliminary pilot tests. All specimens were fabricated under an argon atmosphere with oxygen content maintained below 50 ppm.

**Figure 2:**
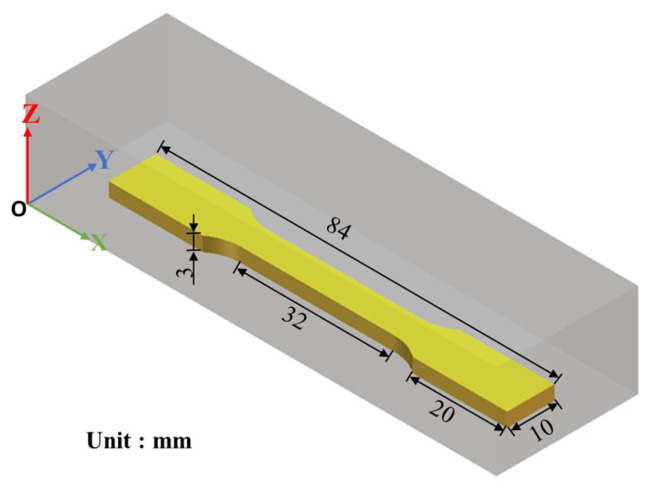
Schematic diagram of a tensile specimen.

A total of 21 cuboid specimens (4 × 4 × 8 mm^3^) were fabricated, with each specimen corresponding to a specific combination of laser power and scanning speed, to evaluate the relative density and establish the processing window. The tensile specimens were designed according to ASTM E8/E8M-16a standard [27], as shown in Figure 2. They were fabricated in a horizontal orientation, such that the building direction (*Z*-axis) is perpendicular to the principal tensile loading axis. Two specimens were tested for each L-PBF condition (as-built and annealed) to ensure reproducibility, and the average values were reported. Annealed specimens were prepared at 675 °C for 6 h, followed by controlled furnace cooling to room temperature (25 °C) [8,28,29]. A cast C95800 alloy bar was used as the control specimen, and the heat treatment conditions were kept consistent.

### 2.3. Characterization

All the specimens for characterization were prepared with the standard metallographic procedures. The XOZ plane of each sample was polished prior to imaging. Optical micrographs were acquired using a Leica DM6M optical microscope (Leica Microsystems, Wetzlar, Germany) at 50× magnification. The relative density of the cubic specimens was evaluated by image processing of ten vertical-section optical micrographs with ImagePro software (Version 6.0.0.260).

The specimens were etched with a solution of 3 g FeCl_3_, 2 mL HCl, and 95 mL distilled water for 20 s to reveal the microstructural features. The microstructure features and the fracture surfaces were characterized using a Nova Nano SEM 450 scanning electron microscope (SEM) produced by FEI Company, Eindhoven, The Netherlands. The phase compositions of the as-built and annealed specimens were examined by XRD using Cu-Kα radiation over the 2θ range of 30–90° at a scanning rate of 1°/min. The Vickers hardness testing was performed by a HVS-1000 tester (Shanghai Shangguang Optical Co., Ltd., Shanghai, China) using a 300 g load applied for 20 s. One representative specimen was utilized for each material state (as-built, annealed, as-cast, and cast-annealed). To ensure data reliability, ten random indentation points were selected on the surface of each sample to obtain the average hardness value and standard deviation. A CMT5504 electronic universal material testing machine (MTS Systems (China) Co., Ltd., Shenzhen, China) was used to conduct the tensile tests with a strain rate of 1 mm/min at ambient temperature. The data of ultimate tensile strength (UTS), yield strength (YS), and elongation (El) were obtained from the tensile curves.

## 3. Results and Discussion

### 3.1. Densification and Defects

**Figure 3:**
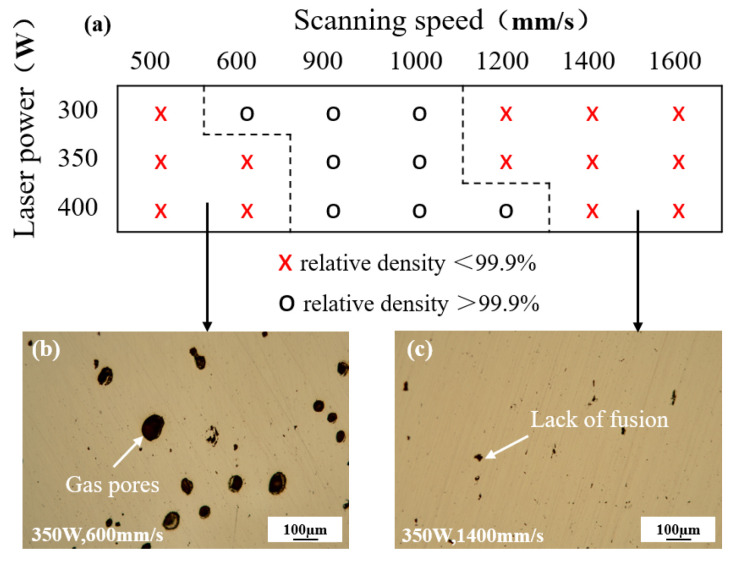
L-PBF forming processing window: (**a**) process map showing the relationship between laser power, scanning speed, and relative density; (**b**) representative OM image showing gas pores formed at high energy input (350 W, 600 mm/s); (**c**) representative OM image showing lack of fusion defects formed at low energy input (350 W, 1400 mm/s).

The relative density results were statistically analyzed to establish the processing window presented in Figure 3. As shown in Figure 3b,c, gas pores are observed at high laser power and low scanning speed, while unfused pores occur at low laser power and high scanning speed. Laser power (*P*) and scanning speed (*V*) directly govern the laser energy input. Excessively high P combined with excessively low V leads to excessive energy input, causing evaporation of the molten metal within the melt pool. The recoil pressure generated by this evaporation intensifies melt pool flow, facilitating gas entrapment and hindering its escape, thus forming gas pores. Conversely, insufficient energy input resulting from excessively low *P* and excessively high *V* prevents complete powder melting and the formation of continuous melt tracks, resulting in irregular unfused pores [30].

**Figure 4:**
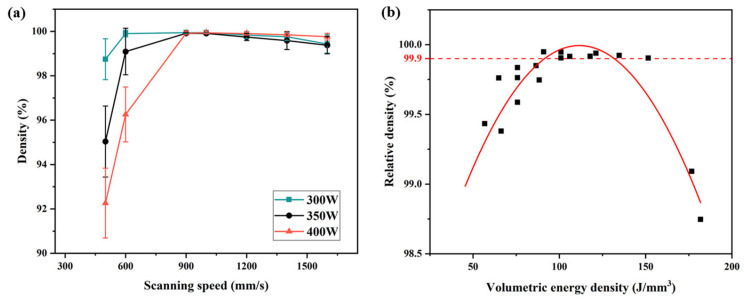
Effect of the scanning speed, laser power (**a**) and VED (**b**) on the relative density for the L-PBFed NAB alloys specimens.

Statistical analysis of laser power and scanning speed reveals their influence on relative density, as shown in Figure 4a. With a constant laser power, the relative density initially increases with increasing scanning speed but subsequently decreases. Considering the combined effects of laser power, scanning speed, hatch spacing, and layer thickness on laser energy input, the volumetric energy density can be introduced to characterize the energy input per unit volume. The VED is calculated as follows [31]:

(1) $$VED=\frac{P}{VST}$$

where *P* is laser power (W), *V* is scanning speed (mm/s), *S* is the hatch spacing (mm), and *T* is layer thickness (mm).

According to Equation (1), the variation in relative density with VED is shown in Figure 4b. Overall, with the increase in VED, the relative density of the specimens initially increases and then decreases. Excessively high or low VED results in gas pores and unfused pores, respectively, as shown in Figure 3b, whereas a suitable VED range (90~135 J/mm^3^) effectively minimizes defects. Notably, most process parameters yielding a relative density > 99.9% fall within this range. Taking into account the density, forming efficiency and stability, the laser power of 300 W and scanning speed of 1000 mm/s were selected to fabricate subsequent specimens for microstructure analysis and mechanical properties testing.

### 3.2. Microstructural Analysis

Based on the densification analysis in Section 3.1, the specimens used for the following microstructural characterization and mechanical testing were all fabricated using the optimized process parameters (Laser Power *P* = 300 W, Scanning Speed *V* = 1000 mm/s, *VED* = 90.9 J/mm^3^), unless otherwise stated.

**Figure 5:**
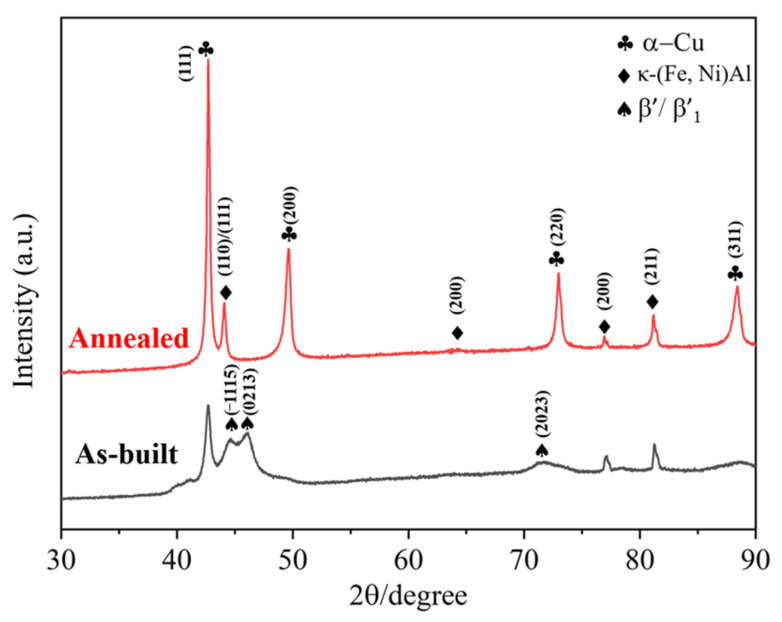
XRD patterns of L-PBF as built and annealed specimens.

Figure 5 shows the XRD patterns of the L-PBF as-built and annealed NAB specimens. The diffraction results confirm the presence of the face-centered cubic (FCC) Cu-rich α-phase matrix and the body-centered cubic (BCC) intermetallic κ-phase (composed of Fe, Ni, and Al). In the as-built condition, the κ-phase diffraction peaks exhibit relatively low intensity. After heat treatment, a noticeable increase in the intensity of these κ-phase peaks is observed. Furthermore, diffraction peaks corresponding to the β′ phase were identified in the as-built samples but were absent after annealing, which is attributed to the eutectoid decomposition (β′ → α + κ_III_) that occurs during annealing [32].

**Figure 6:**
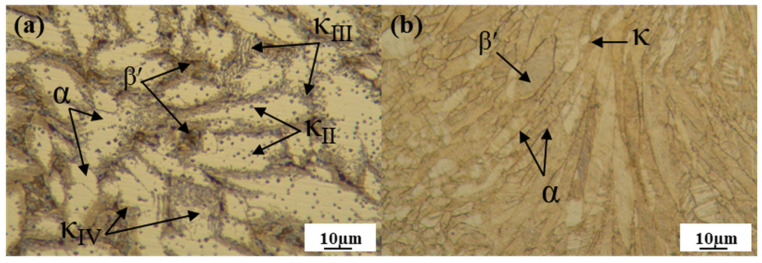
OM diagram of the as-cast specimen (**a**) and the as-built specimen fabricated with optimized parameters (*P* = 30 W, *V* = 100 mm/s) (**b**).

The microstructures of the as-cast and L-PBFed specimens are shown in Figure 6. Significant differences can be observed between the two. It is evident that the grains in the as-cast state are coarser and less uniform compared to those in the as-deposited state. The microstructure of the as-cast specimen mainly comprises a columnar α-phase matrix, accompanied by the precipitation of the β′ phase and intermetallic phases (κ_II_, κ_III_, κ_IV_) [33]. In Figure 6a, the brighter regions represent the α phase and the black iron-rich regions correspond to the β′ phase. κ_II_ appears as irregular spheres; κ_III_ displays a lamellar morphology forming at the α/β′ phase boundaries; and κ_IV_ appears as fine spherical precipitates within the α phase [8,12,15].

The results reveal that the as-built L-PBF specimens primarily consist of columnar β′ grains. Due to the rapid solidification inherent to the L-PBF process, the formation of the lamellar κ_III_ phase is largely suppressed [34]. Consequently, the microstructure features localized α phase along the grain boundaries and minor amounts of fine, globular precipitates dispersed within the β′ matrix, which are identified as the κ_II_ and κ_IV_ phases.

**Figure 7:**
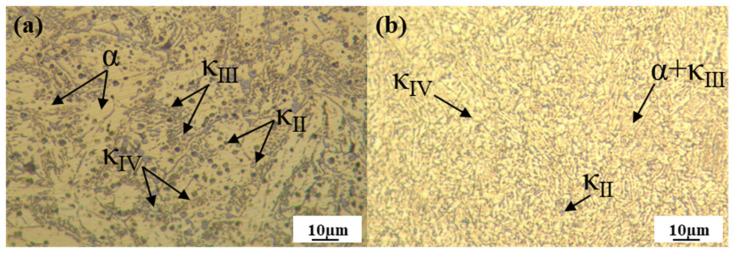
OM diagrams of the annealed as-cast specimen (**a**) and the annealed L-PBF specimen (fabricated at 300 W, 1000 mm/s) (**b**).

The microstructures of the as-cast and as-built specimens after annealing heat treatment are illustrated in Figure 7. Compared to the as-cast state (Figure 6a), the annealed as-cast specimen exhibits the disappearance of the β′ phase, which transforms into a mixture of α and κ phases. Grain refinement and homogenization are observed, with a tendency toward equiaxed growth and a notable increase in intragranular fine phases (iron-rich κ_IV_ phase) [35]. For the as-built specimens, significant microstructural alterations occur post-heat treatment: dendritic structures become markedly more homogeneous, accompanied by a reduction in short and stubby dendritic crystals. A portion of the dendritic crystals gradually evolves into lamellar configurations, some of which already manifest distinct equiaxed characteristics [36]. Concurrently, the β′ phase undergoes substantial attenuation, attributed to its partial transformation into α and κ phases during annealing, consistent with the aforementioned XRD results.

**Figure 8:**
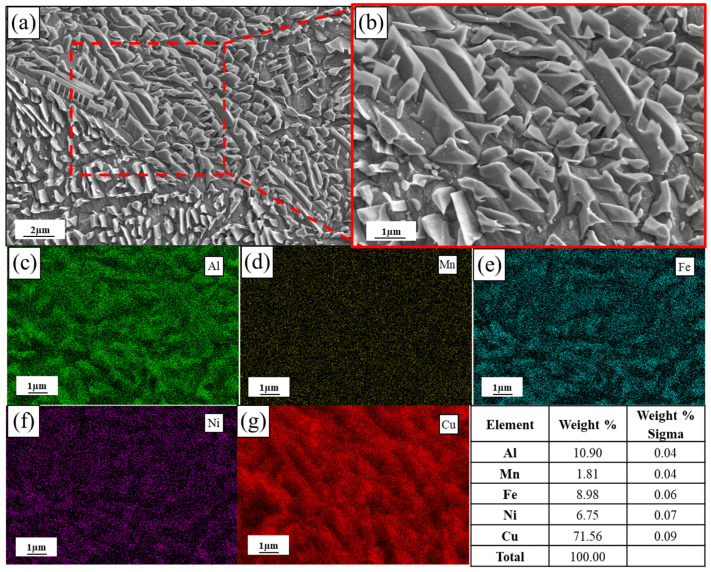
SEM morphology and corresponding EDS maps of the as-built specimen fabricated with optimized parameters (*P* = 300 W, *V* = 1000 mm/s): (**a**) low-magnification SEM image; (**b**) high-magnification SEM image; (**c**–**g**) EDS elemental mapping of Al, Mn, Fe, Ni, and Cu, respectively.

**Figure 9:**
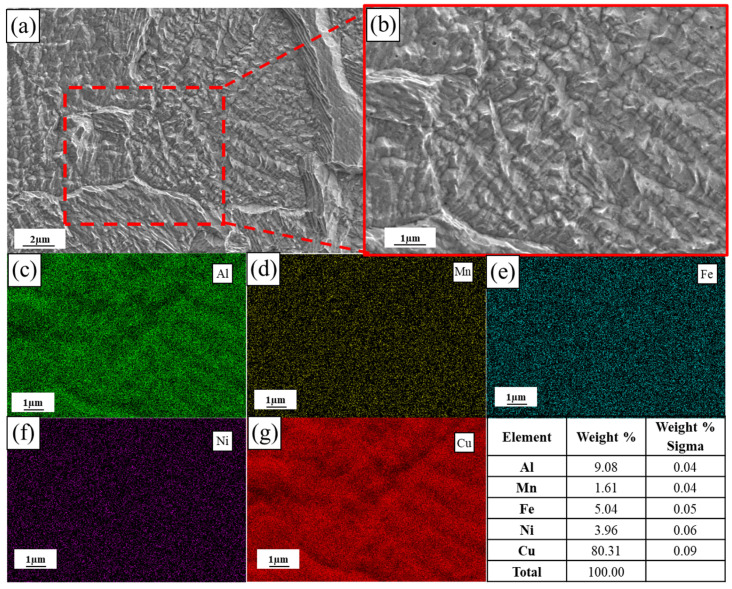
SEM morphology and corresponding EDS maps of the annealed L-PBF specimen (*P* = 300 W, *V* = 1000 mm/s): (**a**) low-magnification SEM image; (**b**) high-magnification SEM image; (**c**–**g**) EDS elemental mapping of Al, Mn, Fe, Ni, and Cu, respectively.

Figure 8a and Figure 9a are the scanning electron microscope (SEM) images of the as-built and annealed state of L-PBF, respectively. Figure 8b and Figure 9b are the corresponding local magnification images and energy dispersive spectrometer (EDS) result images. The distribution of elements in the original state and annealed state was observed by a scanning electron microscope. The EDS element distribution map corresponding to Figure 8b shows that the κ precipitates formed on the β′ phase are rich in Al, Ni and Fe, while Cu is further weakened. In contrast, the EDS element distribution map corresponding to Figure 9b shows that the distribution of alloy elements in the α-Cu matrix after annealing is slightly more uniform; there is no segregation between Fe and Ni, and the distribution of Al elements in the annealed state is not as segregated as in the original state. At the same time, the element content of the κ precipitates rich in Al, Ni and Fe is significantly reduced, while the Cu content increases. This further indicates that the element distribution is more uniform after annealing.

### 3.3. Mechanical Properties

#### 3.3.1. Microhardness

**Figure 10:**
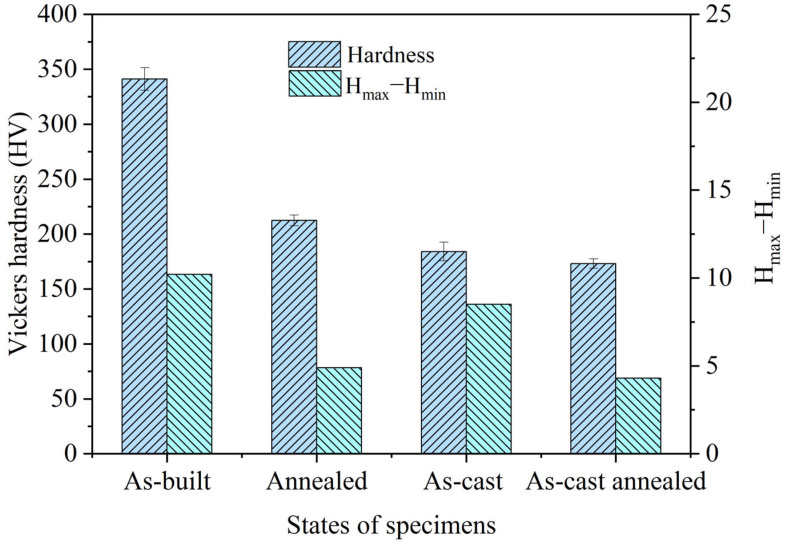
Vickers Hardness of L-PBFed, L-PBF annealed, as-cast and as-cast annealed NAB alloy.

The Vickers microhardness results are presented in Figure 10. The as-built NAB alloy exhibits an average microhardness of 341 HV. This value is significantly higher than that of the as-cast specimen, attributable to the rapid solidification and large thermal gradients inherent to the L-PBF process. These conditions promote the formation of a refined microstructure dominated by a metastable β′ phase (acting as a hard phase) with a fine columnar morphology, consistent with the prior microstructure analysis. Following annealing at 675 °C, a substantial decrease in microhardness is observed. This reduction is primarily attributed to the near-complete decomposition of the metastable β′ phase into an equilibrium α + κ_III_ eutectoid structure during annealing, effectively eliminating the primary strengthening β′ phase. Additionally, the as-built samples display a notable spread between maximum and minimum hardness values, indicating significant microstructural heterogeneity. This heterogeneity likely arises from variations in grain boundary density, layer interface characteristics, and uneven residual stress distribution [37]. In contrast, the annealed specimen exhibits a much smaller hardness range, signifying improved microstructural homogeneity. This improvement stems from stress relief, enhanced elemental distribution uniformity due to diffusion, and potential grain coarsening during annealing, collectively leading to a more uniform hardness profile.

#### 3.3.2. Tensile Properties

**Figure 11:**
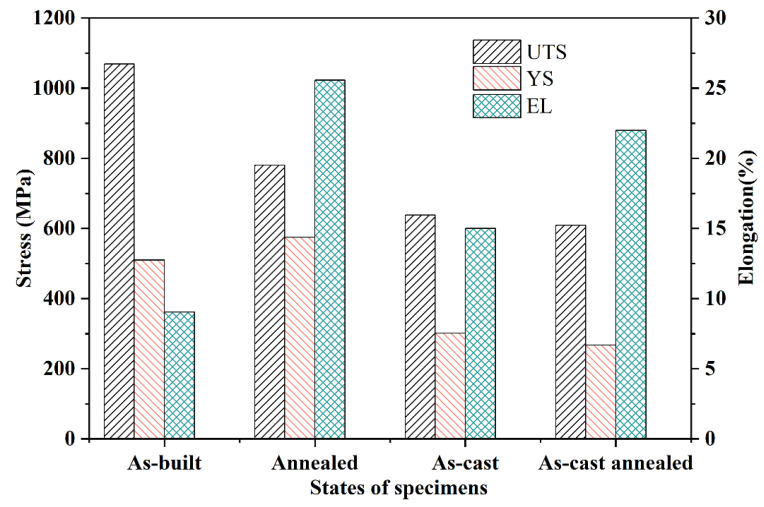
Tensile properties histogram of the L-PBF specimens (fabricated with optimized parameters: 300 W, 1000 mm/s) compared with cast counterparts.

Tensile testing was performed on both as-built and annealed specimens. The tensile properties (UTS, YS, and El) of the as-built and annealed specimens are summarized in Figure 11 and Table 3. Since the mechanical behavior of traditional cast NAB is well-established, this study focuses on the specific performance of L-PBF fabricated alloys. Therefore, standard data for cast and cast-annealed C95800 alloys were adopted from the representative work of Qin et al. [38] to serve as a baseline for comparison. Table 3 summarizes the tensile properties obtained in this work alongside these reference values. Specifically, the reported cast alloy exhibited a YS of 302 MPa, UTS of 638 MPa, and EL of 15%, while the cast-annealed counterpart showed a YS of 268 MPa, UTS of 609 MPa, and EL of 22%. Compared to the as-cast condition, the as-built specimens demonstrated significantly higher YS and UTS but considerably lower EL. This enhancement in strength is attributed to the presence of a fine, columnar-grained microstructure dominated by the metastable β′ phase, which acts as a potent strengthening phase. The refined microstructure results in a high density of grain boundaries, effectively impeding dislocation motion and thereby increasing the yield strength. The inherent brittleness of the β′ phase contributes to the low ductility observed in the as-built state [39].

Following the anneal process, a substantial change in mechanical properties occurred. While the UTS decreased compared to the as-built condition, both the YS and EL exhibited dramatic improvements. Specifically, the annealed specimens achieved a YS of 575 MPa and an EL of 25.6%, representing increases of 12.8% and 184.4%, respectively, over the as-built values. The unexpected increase in yield strength, despite the stress relief and matrix softening associated with annealing, is primarily attributed to the precipitation strengthening effect. During the heat treatment, the metastable β′ phase decomposes, leading to the formation of fine, dispersed κ precipitates (particularly κ_II_ and κ_IV_) within the α matrix. According to the Orowan mechanism, these hard, dispersed precipitates effectively pin the dislocation motion during the initial stages of plastic deformation, thereby raising the yield point [24,39].

Conversely, the significant improvement in ductility stems from two key microstructural changes induced by annealing: (1) The transformation increases the volume fraction of the softer α-phase matrix relative to the brittle β′ phase. (2) The heat treatment promotes a more homogeneous distribution of the κ-phase precipitates within the α-matrix [24]. Furthermore, annealing facilitates elemental diffusion and grain boundary migration, mitigating the micro-segregation observed in the as-built dendritic structure (via EDS). This homogenization delays crack initiation and propagation during plastic deformation, further enhancing ductility.

It is worth noting that L-PBF fabricated components typically exhibit mechanical anisotropy due to the layer-by-layer building process. In this study, the mechanical properties were evaluated using horizontally built specimens (perpendicular to the building direction) to establish a baseline for the optimized parameters and annealing effects. A comprehensive investigation into the full anisotropic behavior (across X, Y, and Z directions) is planned as a future research direction.

#### 3.3.3. Tensile Fracture Morphology

**Figure 12:**
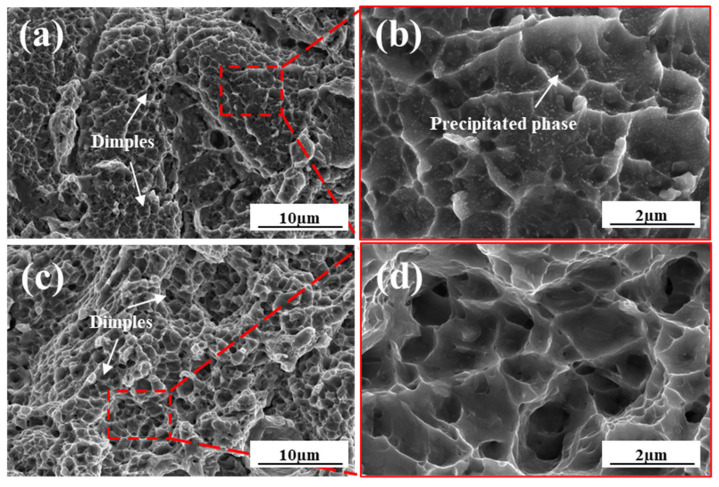
Fracture morphology of tensile specimens fabricated with optimized parameters: (**a**,**b**) as-built state; (**c**,**d**) annealed state.

Fracture surface morphology of the as-built and annealed tensile specimens was examined, as shown in Figure 12. Both specimen groups exhibited predominantly ductile fracture characteristics, evidenced by the presence of numerous dimples across the fracture surfaces. Notably, no distinct cleavage planes were observed in either condition [40], confirming the ductile failure mode. A clear difference in dimple morphology is evident between the two states. As seen in Figure 12a,c, the dimples on the as-built fracture surface are relatively fewer in number, smaller in size, and shallower compared to those observed on the heat-treated specimen. This difference in dimple characteristics directly correlates with the significantly lower elongation measured for the as-built material versus the annealed condition [41]. Furthermore, the magnified view in Figure 12b reveals a higher density of tear ridges within the fracture features of the as-built specimen compared to the annealed specimen shown in Figure 12d. These tear ridges are characteristic features formed during the localized plastic deformation preceding final fracture under tensile loading. The tensile fracture surface of the as-built specimen contains abundant fine precipitates. During tensile deformation, these precipitates impede dislocation motion. They also induce pronounced stress concentration around the precipitate particles. Consequently, the as-built state exhibits relatively low elongation [42].

## 4. Conclusions

Based on the comprehensive analysis of densification behavior, microstructure evolution, and mechanical properties of Laser Powder Bed Fusion (L-PBF) fabricated Nickel–Aluminum–Bronze (NAB) alloy, the following conclusions are drawn:

1. A VED range of 90~135 J/mm^3^ is critical for achieving near-full densification (>99.9% relative density) in L-PBFed NAB. Excessively high VED (>135 J/mm^3^) promotes gas porosity due to melt pool evaporation and turbulent flow, while insufficient VED (<90 J/mm^3^) results in unfused pores from incomplete powder melting. Optimal parameters (300 W laser power, 1000 mm/s scan speed, 0.11 mm hatch spacing, 30 µm layer thickness) effectively minimize defects.
2. Different from the cast specimens, which consist mainly of a columnar α-phase matrix, along with retained β′ phase and intermetallic κ-phase precipitates, due to the rapid cooling rate, the as-built NAB alloy consists primarily of β′ martensite with globular κ particles in the martensitic regions and minor amounts of continuous α phase along grain boundaries. After annealing at 675 °C for 6 h, the metastable β′ phase decomposes into an equilibrium α + κ_III_ eutectoid, validated by XRD peak shifts/disappearance. Elemental homogenization occurs (reduced Al/Ni/Fe segregation via EDS), with κ-phase coarsening along fragmented grain boundaries. Columnar grains partially evolve toward equiaxed morphology, improving microstructural homogeneity.
3. Due to the unique microstructure formed by rapid solidification, the mechanical properties of the as-built samples are significantly improved compared to traditional casting materials: the average microhardness is 341 HV, which is approximately 85% higher than that of the as-cast state. Furthermore, the annealing treatment significantly improves the homogeneity of the microhardness distribution.
4. Annealing significantly enhances the strength-toughness balance: compared to the as-built condition, the elongation increased by 184.4% (reaching 25.6%), while the yield strength (YS) improved by 12.8% owing to the precipitation strengthening effect of the fine κ phases dispersed in the α matrix. Although the ultimate tensile strength (UTS) decreased due to the dissolution of β′ precipitates, it remains higher than that of conventional cast materials. The fracture surface after annealing exhibits uniformly distributed and deeper dimples, which are consistent with the observed improvement in ductility.
5. Limitations and Future Perspectives: Despite the significant improvements in densification and mechanical properties achieved in this study, certain limitations remain to be addressed in future work. Firstly, the mechanical testing was conducted solely on horizontally built specimens; given the layer-wise nature of L-PBF, future investigations will focus on characterizing the anisotropic behavior along the building direction (*Z*-axis). Secondly, while the microstructural homogenization and phase evolution suggest potential improvements in corrosion resistance, electrochemical assessments in simulated marine environments are necessary to fully validate the material’s durability. Nevertheless, the optimized L-PBF NAB alloy demonstrates a superior strength-toughness balance compared to traditional cast counterparts. This combination of properties, coupled with the geometric freedom of additive manufacturing, highlights the material’s significant potential for fabricating complex, high-performance marine components such as propellers, impellers, and valve bodies.

## Figures and Tables

**Table 1:** Chemical composition (wt%) of as-received powder and standard chemical composition of C95800.

	Al	Ni	Fe	Mn	Cu
C95800	8.5–9.5	4–5	3.5–4.5	0.8–1.5	≥79
Powder	9.9	4.3	4.5	1.4	79.9

**Table 2:** Experimental design matrix for the L-PBF processing of NAB alloy.

Experiment Phase	Laser Power (*P*, W)	Scanning Speed (*V*, mm/s)	Volumetric Energy Density (*VED*, J/mm^3^)
I. Process Optimization	300, 350, 400	500, 600, 900, 1000, 1200, 1400, 1600	45.4–181.8
II. Characterization	300	1000	90.9
Fixed Parameters	Hatch spacing = 0.11 mm; Layer thickness = 30 µm; Rotation angle = 67°		

**Table 3:** Comparison of the mechanical properties between L-PBF NAB alloys in this study and cast counterparts from the literature.

Specimen Condition	Yield Strength (MPa)	Ultimate Tensile Strength (MPa)	Elongation (%)	Source
As-built	509.76	1069.38	9.03	This Work
Annealed	574.86	780.11	25.58	This Work
As-cast	302	638	15.1	Qin et al. [38]
As-cast Annealed	268	609	22.0	Qin et al. [38]

## Data Availability

The original contributions presented in this study are included in the article. Further inquiries can be directed to the corresponding author.
